# 4D Printing of Hydrogels: Innovation in Material Design and Emerging Smart Systems for Drug Delivery

**DOI:** 10.3390/ph15101282

**Published:** 2022-10-19

**Authors:** Tuan Sang Tran, Rajkamal Balu, Srinivas Mettu, Namita Roy Choudhury, Naba Kumar Dutta

**Affiliations:** School of Engineering, RMIT University, Melbourne, VIC 3000, Australia

**Keywords:** 3D printing, 4D printing, stimuli-responsive polymers, smart hydrogels, drug delivery

## Abstract

Advancements in the material design of smart hydrogels have transformed the way therapeutic agents are encapsulated and released in biological environments. On the other hand, the expeditious development of 3D printing technologies has revolutionized the fabrication of hydrogel systems for biomedical applications. By combining these two aspects, 4D printing (i.e., 3D printing of smart hydrogels) has emerged as a new promising platform for the development of novel controlled drug delivery systems that can adapt and mimic natural physio-mechanical changes over time. This allows printed objects to transform from static to dynamic in response to various physiological and chemical interactions, meeting the needs of the healthcare industry. In this review, we provide an overview of innovation in material design for smart hydrogel systems, current technical approaches toward 4D printing, and emerging 4D printed novel structures for drug delivery applications. Finally, we discuss the existing challenges in 4D printing hydrogels for drug delivery and their prospects.

## 1. Introduction

Hydrogels are three-dimensional (3D) crosslinked polymer network structures that can absorb and hold a large quantity of water while retaining a distinct shape. Hydrogels can be classified as natural or synthetic (based on the polymer source), physical or chemical (based on the crosslinking type), ionic or non-ionic (based on electric charge), nanoporous or microporous (based on the pore size), nano- or micro- or macro-gels (based on the overall size), and conventional or smart hydrogels (based on stimuli-responsiveness) [1]. Among them, smart hydrogels have recently attracted significant attention owing to their capacity to alter the shape, volume, structure, properties, and functions in response to external stimuli. Smart hydrogels offer several advantages over conventional hydrogels, such as high specificity, good controllability, multi-functionality, tuneability, excellent spatial and temporal resolution, and remote modulation [2]. In the last two decades, smart hydrogels have been extensively studied for healthcare, agriculture, environment, biosensing, tissue engineering, and drug delivery applications [3].

Since the first three-dimensional (3D) printing system was introduced in 1986, the manufacturing industry that adopted 3D manufacturing has undergone dramatic changes; requiring less time, energy, and less waste due to the ability to directly fabricate 3D prototypes from computer-aided designs (CAD) [4]. Over the past several decades, 3D printing technology has made significant progress in healthcare, enabling the fabrication of patient-specific scaffolds/constructs with defined features [5]. It is a highly effective approach for fabricating three-dimensional hydrogels with precise control over their shape and size, structure, and morphology for use in tissue engineering and drug delivery applications.

**Figure 1:**
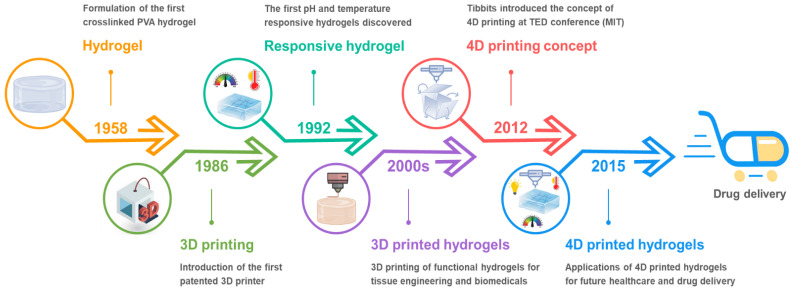
A brief history of the 4D printed smart hydrogels for drug delivery applications. Created with BioRender.com.

Currently, the majority of 3D printed biomaterials/hydrogels for healthcare applications are static and unable to change/transform in response to dynamic changes in the body’s internal environment and biological cues [6]. Advances in dynamic materials, which have the capability to respond to external stimuli over a certain period of time, have opened up new dimensions for engineering future healthcare products [7]. While 3D printing technology has revolutionized the modern manufacturing sector, it becomes even more advantageous when “time” is incorporated as the fourth dimension. With this extra dimension, 3D printed objects can change shape on their own in response to external stimuli such as light, heat, electricity, magnetic fields, and so on [8,9]. 3D printing of such time-dependent, programmable, and intelligent dynamic materials is referred to as 4D printing [10]. Compared to the traditional 3D printed hydrogels, the 4D printed systems can interact with the surrounding environment by responding to stimuli with various outputs including shape-morphing, mechanical motions and biological responses [11]. As a result, 3D printed smart hydrogels (or 4D printed hydrogels) have piqued a surge in interest from both academia and industry for various applications. A brief history of the voyage from the first crosslinked polymer network to the 4D printed hydrogels for drug delivery applications is illustrated in Figure 1.

**Figure 2:**
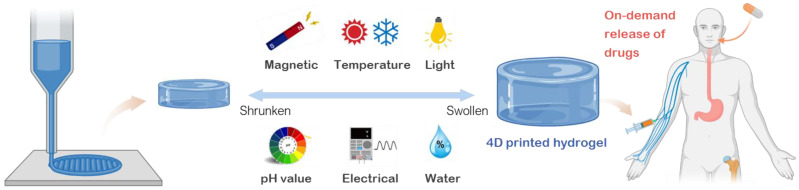
An overview of stimuli-responsive hydrogels and their applications for 4D printed drug delivery systems. Created with BioRender.com. Adapted with permission from [2]. Copyright © 2019 Springer Nature.

Although the research on the traditional 3D printed hydrogels has shown significant growth over the last few years, the development of 4D printed hydrogels is still in its infancy. This review aims to provide an overview of the state-of-the-art 4D printable hydrogel systems and their applications for on-demand drug delivery (Figure 2). First, the development of commonly used stimuli-responsive hydrogels is discussed in correlation with the type of stimuli to which they respond. Second, different technical features of 4D printing techniques are elaborated in detail. Third, the applications of 4D-printed smart hydrogels in drug delivery are explored. Finally, challenges of 4D printed hydrogels are identified and future trends are envisioned. The goal of this review is to stimulate research interests, inspire new novel ideas for 4D printed smart hydrogel systems, and foster the advancement of 4D printing technology for future applications.

## 2. Material Design: Development of Smart Hydrogels for Drug Delivery

Since the description of the first crosslinked hydrophilic polymer more than 50 years ago [12], the systematic design of hydrogels has advanced from static, bioinert hydrophilic polymer networks to dynamic, bioactive hydrogel systems capable of directing specific biological responses such as cellular ingrowth during wound healing and on-demand drug delivery [13].

Within the human body, the biological systems continuously adapt and respond to the dynamic surrounding environments and biological cues. This sophisticated adaptability is accomplished by perceiving and responding to signals like light fluctuations, daily temperature, or biochemical traces. Thus, a necessary initial step in creating smart hydrogel systems for healthcare is to understand the synthesis and processing of constituent hydrogel materials that can expand or contract in response to a variety of stimuli. Based on the kind of external stimulus required for on-demand actuation, we may categorize various stimuli-responsive hydrogels into six major groups, including heat (temperature), magnetic fields, electrical voltage/current, light, pH of the media, and water [2]. The responsiveness and manipulation of hydrogel design under these stimuli opens the door to the development of a variety of 4D printed hydrogels for targeted therapeutic applications. This section highlights aspects of the development of stimuli-responsive biomaterials by analyzing the mechanisms used to transform hydrogel structures when designing dynamically responsive smart hydrogels feasible for 4D printing.

### 2.1. Thermo-Responsive Hydrogels

**Figure 3:**
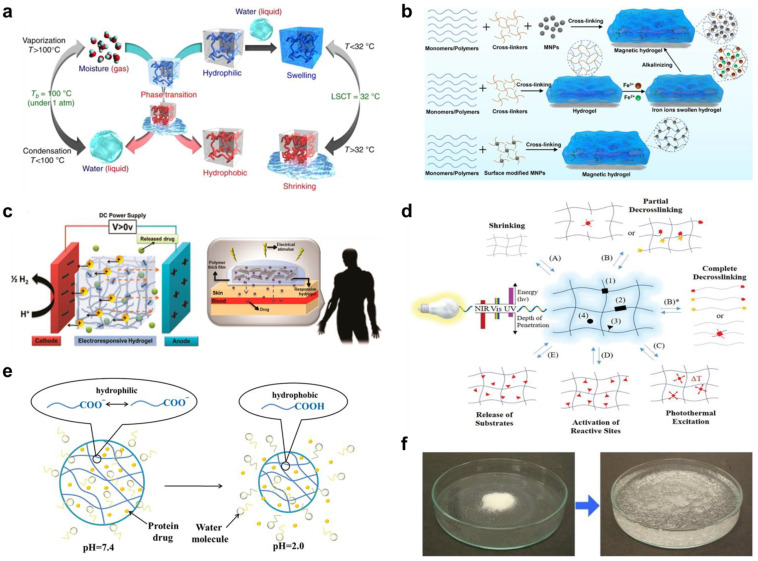
(**a**) Schematic diagram of thermal-responsive hydrogels. Reproduced with permission from [46]. Copyright © 2018 Springer Nature. (**b**) The preparation methods for magnetic-responsive hydrogels. Reproduced with permission from [40]. Copyright © 2021 Elsevier. (**c**) The mechanisms for electro-induced gel deswelling for drug delivery. Adapted with permission from [47]. Copyright © 2015 American Chemical Society. (**d**) Molecular architecture and responses of a photo-responsive hydrogel. Reproduced with permission from [48]. Copyright © 2019 Springer Nature. (**e**) Schematic illustration of pH-responsive hydrogels. Reproduced with permission from [49]. Copyright © 2019 Progress in Chemistry. (**f**) Dry SAP powder and swollen SAP hydrogel. Adapted with permission from [50]. Copyright © 2016 Elsevier.

A temperature fluctuation, particularly in the 36–38 °C range that corresponds to the human body, or ambient settings, is an enticing stimulus for controlling transformer hydrogels. Thermo-responsive hydrogels exhibit temperature-dependent phase behavior and can undergo a sharp sol-gel transition at a critical temperature [14,15,16]. There are two types of thermo-responsive hydrogels: lower critical solution temperature (LCST) hydrogels, which shrink with the rising of temperature above a critical point, whereas upper critical solution temperature (UCST) is the upper bound to a temperature range of partial miscibility. Above UCST the hydrogel systems are in an expanded or swollen state (Figure 3a). 3D printing of such thermo-responsive hydrogels is an attractive route to create 4D printed hydrogels, which are conceivable to the temperature of the surroundings, for on-demand drug delivery applications.

The literature on LCST hydrogels is dominated by poly(N-isopropyl acrylamide) (pNIPAM) and its derivatives, because of its massive volume change at a relatively low critical temperature of around 32 °C [17,18,19]. The biocompatibility and ease of processing of pNIPAM are demonstrated in a recent work by Allen and co-workers [20] by culturing 3T3 fibroblasts on cell sheets produced from aligned electrospun fibers, demonstrating the promising prospect of pNIPAM for 4D printing toward drug delivery applications. Thermal responsive pNIPAM-based hydrogels with tunable responsiveness (critical temperature and swelling characteristics) have been developed by adjusting the number of repeating monomer units in (oligoethylene glycol methacrylate) (OEGMA) [21], or by combining it with polymers such as poly[di(ethylene glycol) ethyl ether acrylate] (PDEGA) [22]. In contrast, UCST hydrogels expand as the temperature rises. This positive thermal responsivity broadens the design space for future smart hydrogels. The most frequently employed UCST hydrogels are interpenetrating networks of polyacrylamide (pAAm) and polyacrylic acid (pAAc) [23,24]. Recent research also showed that smart biomaterials, such as highly elastic protein elastic and elastin-mimetic proteins; resilin and resilin-mimetic proteins can be designed to exhibit tunable LCST and UCST transitions in physiological solutions [25,26]. Extensive research by Dutta et al. and others [25,27,28,29] has demonstrated that resilin and resilin-mimetic proteins can exhibit tunable multi-stimuli-responsiveness including both LCST and UCST. 

Significant research efforts have also been focused on enhancing the biocompatibility and biodegradability of thermo-responsive hydrogels. Ye and co-workers [30] have developed a supramolecular UCST hydrogel for sustained-release drug administration and tissue engineering scaffold using polyglycerol sebacate (pGS), a novel biodegradable elastomeric material with outstanding biocompatibility. Greater biodegradability can be achieved by the use of hydrolytically and enzymatically labile bonds [30], or by introducing biodegradable monomers like benzomethylene dioxepane or methacrylate polylactide into their polymeric backbone [31,32]. Natural polymers such as gelatine, cellulose, and chitosan can also be functionalised with poly(L-alanine-co-L-phenylalanine), poly(ethylene glycol), and glycerol phosphate to form smart hydrogels that are both thermal responsive and biodegradable [33,34], promising for 4D printed drug delivery systems.

### 2.2. Magnetic Responsive Hydrogels

The magnetic field has also been studied as a potential external trigger for controlling the properties of smart hydrogels. The ability to activate remote actuation with a fast response time and biocompatibility even at high field strength makes electromagnetic a favorable stimulus, especially in vivo applications. To achieve magnetic responsiveness in hydrogels, exogenous additives like paramagnetic or ferromagnetic are included in the polymeric matrix, allowing for rapid and large actuation behaviors in response to magnetic fields [35]. Magnetic additives such as metal alloys (e.g., iron and neodymium alloy), metal oxides (e.g., ferrous ferric oxide), and functionalized magnetic nanoparticles can be coupled with pNIPAM, pAAm and gelatin in a chemically or physically crosslinked polymer network to form magnetically responsive smart hydrogels [36,37,38]. More complex magnetic responsive hydrogels with stronger interactions between the magnetic particles and the polymer network were also created via covalent and coordination bonds [39]. These hydrogel systems, in general, do not require extra crosslinkers during synthesis and gelation occurs spontaneously when mixed. The preparation methods for magnetic responsive hydrogels are illustrated in Figure 3b [40].

There are two main action modes in magnetic responsiveness in hydrogels for controlled drug release, that are changing the direction of the magnetic field to arrange perpendicularly or parallelly to the drug diffusion direction and switching on/off the magnetic field to trigger release [41,42,43]. Several magnetic responsive smart hydrogels are currently being tested in vivo in animal models and have the potential to translate to clinical drug delivery applications [44,45]. 

### 2.3. Electrical Responsive Hydrogels

Inspired by artificial muscle biomimicry, electrical responsive hydrogels can expand or contract under a solvent-induced or an externally applied electrical field [51,52]. The use of an external electrical field as a stimulus has particular advantages for drug delivery due to quick, precise, and programmable responsiveness [53,54].

For the controlled release of drug, electrical responsive hydrogels are fundamentally based on the mobility of ions in response to an electrical field and the rearrangement of the ion concentration profile at the hydrogel-swelling media interface. Equilibrium is achieved through the balance of fixed charges on the polymer backbone and counterions attracted by the surrounding swelling media. As a result, the ion concentration is not uniformly distributed inside and outside the gel, creating an osmotic pressure that causes swelling or deswelling (Figure 3c) [47].

Electrical responsive hydrogels are typically polyelectrolytes with ionizable groups along their side chains or polymeric backbone [55,56]. Numerous synthetic polyelectrolytes and related copolymers have been utilized to fabricate electrical responsive hydrogels, including poly (vinyl alcohol) (PVA) [57], poly(sodium maleate-co-sodium acrylate) [58], PVA/pAAc [59], pAAc/poly(N-vinylpyrrolidone) [60], and sulfonated polystyrene (s-PS) [61]. There are also natural polyelectrolytes, including proteins [62,63], polysaccharides [64], and polypeptides [65], that respond to electrical stimuli [66,67,68]. They can be combined with synthetic polymers to create hybrid electrical responsive hydrogels, for instance, fibrin protein blended with pAAc [69], chitosan coupled with poly(N,N-dimethylacrylamide) [70], and alginate combined with poly(methacrylic acid) [71] for 4D printed drug delivery systems.

### 2.4. Photo-Responsive Hydrogels

The use of light as a stimulus is particularly advantageous for remotely inducing the expansion and contraction of 4D printed hydrogels for controlled delivery of therapeutic agents. The two primary mechanisms of photo-responsive hydrogels are based on reversible crosslinking and photothermal excitation (Figure 3d) [48]. Both approaches can be accomplished by including photoactive moieties into the hydrogel matrix [48]. For reversible crosslinking, the presence of photoactive moieties such as azobenzene or o-nitrobenzyl groups can induce the photocleavage or photoisomerization of hydrogel matrices upon illumination, resulting in reversible contraction–expansion of polymer chains [72,73]. A second way to achieve light-induced deformation is by employing photothermal nanomaterials, which rapidly convert light irradiation to heat dissipation, to control the reversible dehydration–hydration processes of the photo-responsive hydrogels [74,75]. 

Numerous nanomaterials, including inorganic nanomaterials (e.g., gold and neodymium oxide) [76], carbon-based materials [77], and black phosphorus [74], have been introduced into photo-responsive hydrogels. Researchers have demonstrated photoresponsivity by incorporating gold nanorods into pNIPAM-AAc hydrogels [78], others reported smart hydrogels (agarose and pNIPAM) containing single-walled nanocarbons (SWNTs) and single-walled nanohorns (SWNHs) that show marked phase transitions upon NIR irradiation [79,80]. Additive manufacturing of such intelligent photo-responsive hydrogels is still in its infancy yet promising for 4D printed drug delivery systems.

### 2.5. pH Responsive Hydrogels

Apart from physical stimuli, physiological conditions (e.g., the inherently low pH of the stomach, or the slightly alkaline condition of the blood) have been exploited to initiate swelling-controlled drug release from hydrogel carriers [81]. Systems that are capable of responding to a dynamic pH environment are useful for healthcare applications, as various places throughout the human body experience pH variations during a disease condition. The dynamic pH ranges in various tissues and cellular compartments in the human body are detailed in Table 1 [82].

The pH sensitivity of a hydrogel network can be modified by adjusting the hydrophilicity and ionic character of the internal pendant functional groups (Figure 3e) [49]. It has been demonstrated that hydrogels containing acidic moieties swell as the acid groups deprotonate at higher pH. Cationic groups, on the other hand, generate more swelling at lower pH values [83]. Thus, there are two major types of pH-responsive hydrogels: anionic and cationic hydrogels. Anionic hydrogels contain pendant groups that ionize at a pH greater than their acid dissociation constant (pKa) and expand at higher (primarily basic) pH values. Due to the presence of physical interactions between the polymer chains, their polymer networks remain folded at pH values less than their pKa (low-pH environment). Conversely, cationic hydrogels expand when the pH level falls below pKa and contract when the pH value rises over pKa [84]. 

Anionic hydrogels are frequently constructed of crosslinked polymer networks containing carboxyl groups (polyacrylic acid [85], polymethacrylic acid [86], and polycarboxymethyl agarose [87]) and their copolymers [88]. Monomers bearing amine and amide groups, such as AAm [89], dimethylaminoethyl methacrylate (DMAEMA) [90], and 2-(diethylamino)ethyl methacrylate (DEAEMA) [91], as well as their copolymers [92], are commonly used as constituents of cationic hydrogels. Emerging hydrogels made of biopolymers such as alginate [93], gelatine [94], chitosan [95], and albumin [96], resilin-mimetic proteins [68], silk [97], soy protein [98], and their blends and composites could also exhibit pH responsiveness with superior biocompatibility and biodegradability compared to their synthetic counterparts. With the dynamic pH ranges in various tissues and cellular compartments in the human body, 4D printed pH-responsive hydrogels are advantageous for precisely controlled drug release into a specific area of the human body under a specific condition.

### 2.6. Water Responsive Hydrogels

Water responsive hydrogels, or superabsorbent polymers, are crosslinked three-dimensional interconnected macromolecular networks possessing extremely high liquid swelling capacity (Figure 3f), providing an effective vehicle for therapeutic agents to be encapsulated and released in biological environments. Water-responsive hydrogels are often composed of ionic monomers and are weakly crosslinked. As a result, they exhibit an extraordinary capacity for water absorption [99,100]. Water-responsive hydrogels have been the most commercially successful members of the hydrogel family and are widely used in healthcare products, including pharmaceuticals, personal hygiene, wound dressing, and drug delivery applications [101,102].

At present, the majority of water responsive hydrogels are mostly synthetic or petrochemical in origin, and they are predominantly composed of acrylic monomers, most frequently acrylamide (AAm) [103], acrylic acid (AAc) [104], and their copolymers [105]. In recent years, the trend towards substituting “greener” alternatives in water responsive hydrogels is more and more pronounced due to the low degradability and biocompatibility of the synthetic incumbents. As a result, emerging bio-based water responsive hydrogels are being made from renewable raw materials such as cellulose [106,107], soy protein [108,109], starch [110], natural gums [111], chitin [112] and their hybrids and composites, providing a customizable and effective route toward 4D printed hydrogels for drug delivery applications.

## 3. Technical Approaches toward 4D Printing of Hydrogels

While conventional 3D printing produces static hydrogels, 4D printing strategies are based on the concept of integrating time-dependent smart materials to 3D printing, whereby the printed hydrogels can facilitate transformation over time under stimulation. Therefore, technical approach toward 4D printing is an extension and inseparable from 3D printing in every aspect, including its technical and design perspectives.

### 3.1. Printing Techniques for Smart Hydrogels

**Figure 4:**
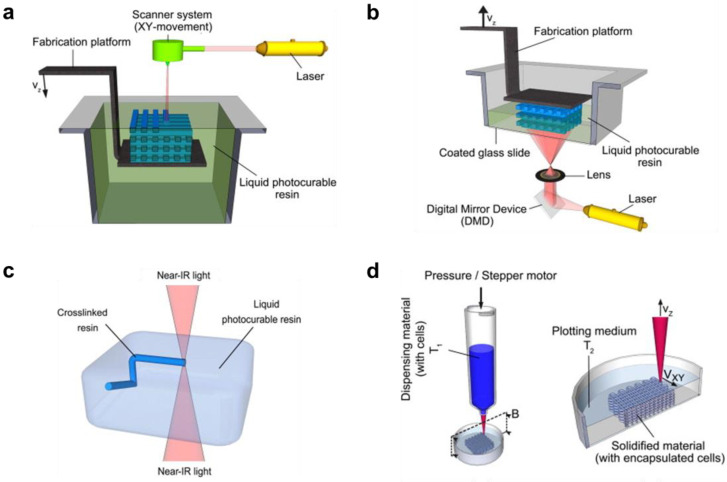
Schematic of (**a**) stereolithography, (**b**) digital light processing, (**c**) two-photon photopolymerization, and (**d**) extrusion 3D bioprinting. Adapted with permission from [118]. Copyright © 2012 Elsevier.

Smart hydrogel systems for drug delivery can be 3D printed or 3D bioprinted (with cells) from hydrogel-forming solutions (inks comprising crosslinkable, biocompatible, and stimuli-responsive monomers or polymers) using the laser-based or extrusion-based techniques [113]. The laser-based techniques include: stereolithography (SLA), digital light processing (DLP), and two-photon polymerization (2PP); whereas the extrusion-based techniques include plotting (solution-based) through a nozzle (Figure 4). In SLA, photocurable polymer solution (resin) is held in a container (bath) and the 3D structures are cured one layer after another by horizontal plane movement (X- and Y-directions) of spot-promoting a laser beam (usually UV light) on the vertically moving (Z-direction) fabrication platform (Figure 4a); whereas, DLP (Figure 4b) uses a projector to illuminate a specific area [114]. In 2PP (Figure 4c) ultrashort laser pulses from a near-infrared femtosecond laser source are focused within a small volume in the photocuring polymer solution, where a suitable photoinitiator within the resin can simultaneously absorb two photons of 800 nm wavelength and let them serve as one photon of 400 nm wavelength (UV light region) to trigger localized polymerization of 3D structures in the bath without affecting other areas [115]. In extrusion printing (Figure 4d), direct-ink-writing (DIW) or fused deposition modelling (FDM), a polymer solution with controlled viscoelastic behavior is extruded (pneumatically or screw-driven) through a nozzle to create a layer-by-layer added 3D structure [116]. Some curing and stabilization strategies are usually required during or following the printing procedure [117].

### 3.2. Crosslinking Strategies for the Fabricated 4D Printed Hydrogels

**Figure 5:**
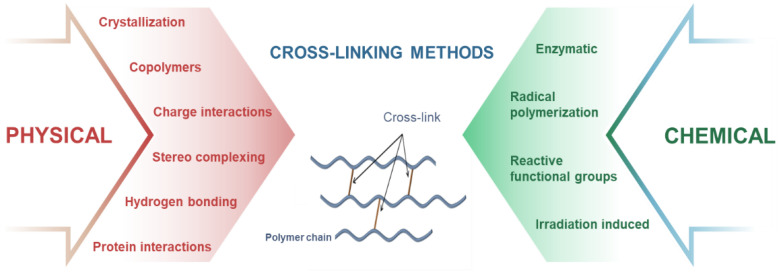
Different methods of crosslinking hydrogels.

In correlation with the printing techniques, crosslinking of the extrudable inks is an essential step toward printing of multilayer and complex architectures. Smart hydrogels can be fabricated from stimuli-responsive monomers and polymers by various physical and chemical crosslinking strategies [119], which can be utilized as useful tools for 4D printing (Figure 5). In crystallization strategy, crosslinking of the printed structures is achieved by repeated freeze–thaw cycles, which causes nucleation and growth of microcrystals in the structure that act as physical crosslinking sites leading to gelation. Freeze–thawing has been widely used for crystallization of highly elastic PVA hydrogels [120], which can be employed for 4D printing of hydrogels. For copolymers strategy, printable inks comprising both hydrophilic and hydrophobic units are formulated by attaching hydrophilic or hydrophobic fragments to polymer chains, which can aggregate in water to form hydrogels by self-assembly of hydrophobic segments. By grafting NIPAAm onto chitosan, Kim et al. [121] developed thermo- and pH-responsive hydrogels, which are 4D printable. In charge interaction strategy, in situ crosslinking between oppositely charged molecules within the inks is achieved by pH changes, which cause ionization or protonation of ionic functional groups and induce gelation. As demonstrated by Prado et al. [122], a novel pH responsive interpolyelectrolyte complex based on two natural polysaccharides (cationized starch and kappa-carrageenan, as counterion) has been prepared for use as matrix for controlled drug release, laying the foundation for developing 4D printed hydrogels for drug delivery. Another strategy is utilizing the interactions with the hydrogen bonds, where gelation occurs due to the aggregation of the printed inks by regeneration of hydrogen bonds between molecules. For example, You et al. [123] prepared a new hydrogel with a hierarchical hydrogen bond system consisting of weak hydrogen bonds between N,N-dimethylacrylamides (DMAA) and acrylic acids (AAc), and strong multiple hydrogen bonds between 2-ureido-4[1H]-pyrimidinone units. This crosslinking strategy can be coupled with DIW for 4D printing applications. In the stereo-complexing strategy, gelation of the printed inks occurs due to interactions between polymeric chains or small molecules of the same chemical composition but with different stereochemistry. By grafting L-lactide and D-lactide oligomers to dextran, which can be 4D printed and induced spontaneous gelation in water, Jong and colleagues [124] developed smart dextran-based hydrogels for drug release. For protein interactions strategy, gelation of the printed inks is induced by peptide sequences with specific physicochemical properties. For example, Petka et al. [125] developed pH- and temperature-responsive reversible hydrogels using artificial proteins synthesized by the recombinant DNA method. The printable protein formulations consist of terminal leucine zipper domains flanking a central, flexible, water-soluble polyelectrolyte segment, which forms coiled-coil aggregates of the terminal domains in near-neutral aqueous solutions and triggers the formation of a three-dimensional polymer network.

For chemical crosslinking, radical polymerization and irradiation-induced crosslinking strategies are intensively used for 3D/4D printing, where gelation occurs due to the generation of reactive species by ionization (usually UV or electron-beam irradiation), which leads to co-polymerization (between polymer chain and grafting monomer), and crosslinking reaction. As a typical example, Amin et al. [126] developed thermo- and pH-responsive bacterial cellulose/acrylic acid hydrogels by accelerated electron-beam irradiation, which led to the curing of acrylates. This ink formulation can be coupled with electron-beam irradiated extrusion printing for fabricating 4D printed hydrogels. By exploiting reactive functional groups within the ink formulation, crosslinking of the printed structures occurs due to covalent reactions (addition, condensation, Schiff bases, and click-type) between the functional groups of the polymers (mainly hydroxyl, carboxyl, and amino groups) that provide solubility to water-soluble polymers. Using the same strategy, Tortora et al. [127] prepared PVA-based hydrogels by Michael-type addition reactions, applicable for 4D printing. On the other hand, in the enzymatic method, crosslinking of the inks occurs by the formation of covalent bonds between a free amine group and the γ-carboxamide group of polymers (by transglutaminase enzyme), or between phenol and the hydroxyl group of polymers (by tyrosinase enzyme), etc. As a typical example, Chen et al. [128] prepared chitosan and gelatin crosslinked hydrogels catalyzed by transglutaminase and tyrosinase. This strategy is compatible with DIW for 4D printing.

### 3.3. Design Considerations for 4D Printing

The design considerations for 3D printing of static hydrogels have been extensively reviewed [129]. However, there is scant literature on 4D printing of hydrogels with the ability to dynamically change shape and properties on demand. As 4D printing is an extension of 3D printing in many aspects, in this section, we discuss the design considerations for 3D printing and the concept of integrating time-dependent smart materials to elucidate the design considerations for 4D printing. 

**Figure 6:**
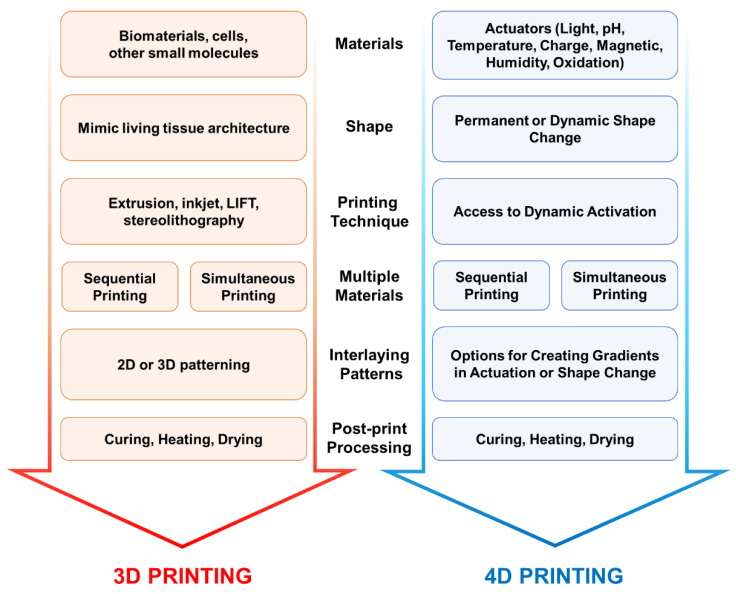
Design considerations for 4D printing of smart hydrogels.

*Materials*: For 3D printing, the first major consideration that needs to be addressed is the composition of the materials to be used in the hydrogel ink preparation (Figure 6). The hydrogel-forming materials (monomers, polymers, crosslinkers, rheology modifiers), the cells and other biomolecules that sustain the growth of the living cells in the 3D printed bio-construct are part of the bio-ink. The hydrogel formulation may contain synthetic/biobased/biomimetic single or multiple monomers. The hydrogel formulations without the cells are defined as biomaterial inks that can be used to 3D print scaffold materials for cell growth [130]. The biomaterial inks with the inclusion of cells are defined as bio-inks. The cell types used in the ink can be multiple depending on the biological complexity of the 3D printed living construct. For example, in the case of printing a human intestine architecture, the living construct can be printed layer by layer manner, where the hydrogels are printed first, followed by epithelial cells on top of them, and then the microvilli structure is printed on top. To increase the biological complexity of 3D printed living intestine and build a realistic intestinal environment, the other original human cell types such as enterocytes, tuft, goblet (mucus-secreting), paneth cells and stem cells can be printed in an interlacing manner. For bioinks to be acceptable for tissue engineering applications, they must strike a balance between biocompatibility and printability, known as the bio-fabrication window. The sequential or simultaneous printing methods need to be considered if multiple materials need to be printed, as shown in Figure 6.

In the case of 4D printing, the actuators that induce a smart response to the 3D printed living construct need to be introduced into the inks. The actuators can be either added while printing such as magnetic, electronic, and oxidative properties by the incorporation of materials that achieve it or post print actuators such as light, pH, temperature, and humidity, etc. can be used on appropriately responsive gels. This additional material consideration for 4D printing is crucial as the addition of them may significantly affect the rheological and processing characteristics, hence the printability of hydrogel ink.

*Shape*: The second design consideration involves the shape of the 3D printed structure. One of the key criteria for 3D printing a living construct is that it should realistically simulate the microarchitecture of the organ or biological construct that is being printed. In some cases, the secondary nanostructures may need to be introduced by post-printing. 

The shape of the 3D printed structure before and after the application of the actuator needs to be considered very carefully for 4D printing case. The actuator (stimuli) once applied changes the 3D printed construct significantly. Hence, in the printed design there should be enough room for the structure either to expand or contract without compromising the strength and other physical properties of the hydrogel. The appropriate simulations must be carried out prior to the printing to check the effect of stimuli on the shape hence its structural integrity. Another important consideration is whether the change induced by the stimuli is permanent or transient.

*Printing Technology*: The selection of printing method is a critical consideration as it determines the resolution and the materials that can be used in the printer. As reviewed extensively in the literature [129,131] and in the current article, stereolithography, digital light processing, two-photon photopolymerization, laser-guided direct writing and extrusion-based printing techniques are popular options. The resolution of printing may vary from hundreds of nanometers to hundreds of microns depending on the printing technique employed. The resolution of the printing technique should be on the order of the smallest feature that needs to be accurately captured in the living construct.

As mentioned earlier, the selection of printing method is a critical consideration, especially in the case of 4D printing. For creating stimuli response hydrogels, many times the creation of material gradients is necessary. The gradients in either the material properties or structures would help control the shape change behavior of the printed construct. Hence, during the selection of an appropriate print method, it is critical to consider the option to have multiple printheads that can create gradients in material properties.

*Interlaying Patters*: As discussed earlier, in the case of printing a living human intestinal architecture, we discussed the printing of multiple cell types in the 3D constructs. However, to simulate different regions of the human intestine, the microarchitecture can be varied spatially either in 2D (on the surface of the printed construct) or in 3D (inside the construct). The gradients in the spatial structure can be 2D or 3D as needed. The interlaying pattern shape and size need to be given particular attention as the spacing between the printed structure may decrease or increase when external stimuli are applied. During the construction of the digital file for 3D printing the spacing between the interlaying pattern needs to be adjusted according to the order of magnitude change in the size of the printed structures when subjected to external stimuli.

*Post-Treatment*: The post-treatment of printed construct is essential to improve the strength and physical properties. The post-processing may involve further curing, heating and drying. In the cases where secondary nanostructures need to be added to the existing print structure, plasma processing and other nano-texturing methods (stamping with nano-textured solid substrate under compression or heating) can be used. 

### 3.4. Multi-Materials Extrusion 4D Printing

Multi-materials printing has been a crucial development that resulted in the explosion of 4D printing techniques to fabricate multi-stimuli responsive AM structures [132,133]. There is a difference between the multi-materials formulation versus the multi-materials printing. The key difference is, in the former case; the formulations consist of multiple materials homogenized as a single ink to print using a single nozzle. Here, the multi-material formulation may impart various desired physical and chemical properties to the ink and the printed structure. In some cases, the printed structure may phase separate after printing to provide desired structure and performance. However, in the case of multi-material printing, multiple inks are printed with multiple nozzles in a pre-determined pattern to create an interlacing pattern [134,135]. This interlacing pattern within the 3D printed structure when exposed to a particular stimulus responds in a non-linear fashion resulting in the printed shape morphing into the desired shape.

**Figure 7:**
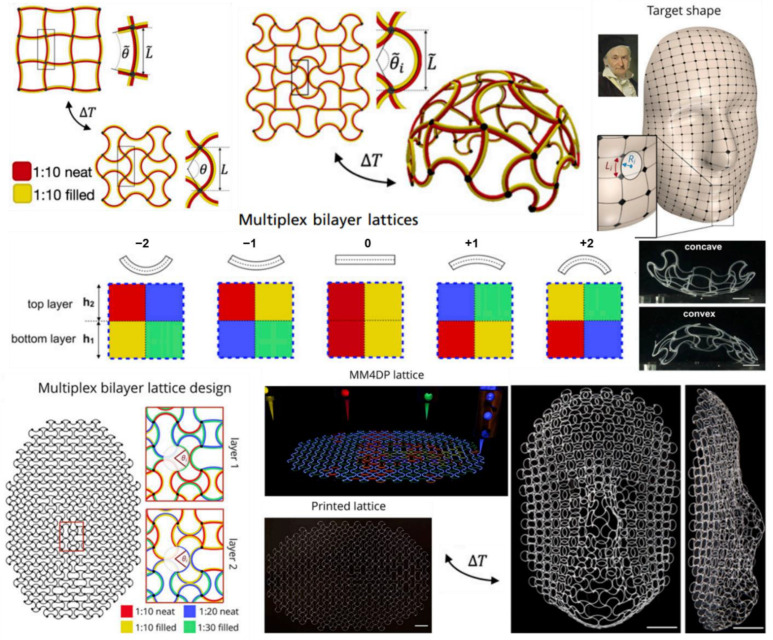
Multi materials lattice printing, where the elasticity and thermal expansion coefficient for the printed lattice is precisely tuned to obtain the final desired form of the printed structure using temperature as external stimulus. Adapted with permission from [132]. Copyright © 2019 PNAS.

Boley et al. [132] have developed a different, multi-material approach for the 4D printing of stimuli-responsive and shape-morphing structures. In this approach for printing multi-material lattices, a multi-material lattice (either single or many layers in Z-direction) is the first 3D printed in a predetermined order before being exposed to external stimuli to morph the lattice into the desired shape (Figure 7). Multiplex bilayer lattice method prints a bilayer consisting of multiple ink formulations to control the curvature of the printed structure to an external stimulus such as temperature. The local curvature for the print lattice is tuned by formulating inks with varying elasticity and thermal expansion coefficient. The resulting curvature of the structure depends on the lattice pattern. The desired shape morphing is achieved locally by varying the bilayer lattice pattern for individual voxels (3D pixels) of the print structure. Such a printing method has also been applied to develop a shape morphing patch antennas that can change the resonance frequencies on the fly with an applied external stimulus.

**Figure 8:**
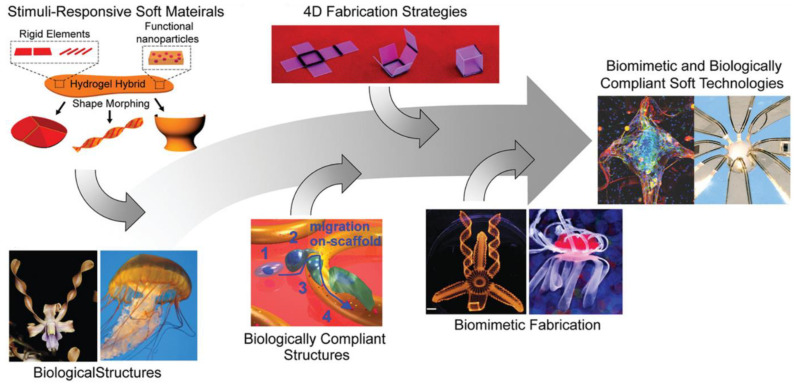
Multi-material 4D printing for biomimetics. Reproduced with permission from [136]. Copyright © 2019 Wiley.

4D printing of biomimetic structures is a fascinating area of research that can explore fundamental nature of control of actuation based on how nature has evolved these structures. The structure mimicking of the nature can teach the research community about the tricks involved in achieving stimuli response by the biological entities. Especially aquatic organisms have evolved complex actuation mechanisms either to evade the predators or to hunt for food. Multi material printing is capable of realizing the biomimicry of aquatic organisms. For example, McCracken et al. [136] have developed multi material print schemes using ionotropic gradient hydrogels to mimic sea-jelly 3D structures that can shape morph when hydrated (Figure 8). The spatial gradients were achieved by selectively programming the valency of the ion-binding agents in multi material printed layers. Spatial gradients in combination with printed geometry, enable the programming of flexibility and movement of iron oxide nanoparticle–loaded ionotropic hydrogels to morph into 4D-printed structures when hydrated.

Anisotropic swelling and elasticity of printed materials were also utilized for achieving biomimetic 4D printing [137]. Shear-induced alignment of cellulose fibrils in the print ink results in anisotropic swelling ratio and elasticity. When multiple layers are printed with a pre-determined pattern, during swelling, the printed 3D structure shape morphs into the desired biomimetic shape [138].

**Figure 9:**
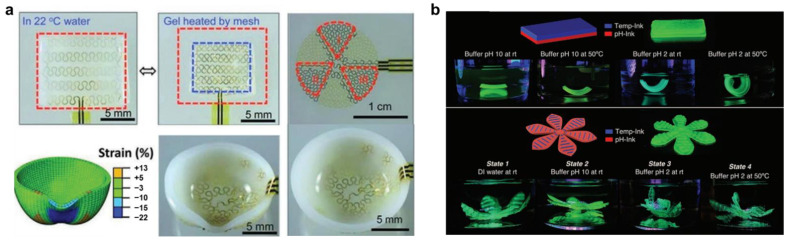
Multi-material 4D printing for shape transformation combining hard and soft materials. (**a**) heating elements are patterned into a soft thermo-responsive hydrogel. Adapted with permission from [139]. Copyright © 2013 Wiley. (**b**) Multi-ink printed hydrogels with different responsiveness such as temperature, pH, and enzymatic response. Adapted with permission from [140]. Copyright © 2021 Wiley.

Another interesting approach for achieving 4D printing is to combine hard and soft materials, where hard material is integrated into a soft hydrogel structure [139]. The hard material microstructure is generally fabricated using conventional photolithographic techniques, which can then be embedded into soft hydrogel materials by casting. The programmable actuation of these structures can be achieved either through heat (Joule Heating Mesh) or piezoelectric effects. Figure 9 shows such an example, where heating elements are patterned into a soft hydrogel that is thermo-responsive with specified LCST. When the gel gets heated by the mesh, the hydrogel contracts when the temperature reaches above LCST. The expansion and contractions are reversible with temperature. In this method, when the heating elements are distributed non-linearly throughout the hydrogel, which can be independently controlled, the underlying hydrogel can be morphed into the desired shape (Figure 9a).

Narupai et al. [140] developed protein hydrogel-based multiple inks with temperature, pH and enzymatic responsiveness. When these inks were printed in combination, the resulting 3D structures shape morphed when subjected to the respective stimuli (Figure 9b). These kinds of protein-based hydrogel have potential for interesting applications for wound healing for external as well as internal organs. Summarily, a wide range of concepts, materials and processes have been developed for intelligent manufacturing using 4D printing, which has the potential to revolutionize the way the product is being manufactured and it is in its infancy.

## 4. Emerging 4D Printed Hydrogels for Drug Delivery

Drug delivery refers to the development of novel materials or carrier systems for the effective therapeutic delivery of drugs. Hydrogels are attractive candidates for drug delivery applications as they can provide spatial and temporal control over the release of various therapeutic agents, including chemical drugs, small biomolecules, and cells. Hydrogels swell fast in aqueous environments like body fluids, which could lead to burst (in a short period) release of drugs, particularly for small molecules below the size of the hydrogel mesh size. Therefore, drug–polymer interactions are essential for the controlled release of drugs from hydrogels. Various physical and chemical interactions, including electrostatic, hydrophobic and covalent, conjugation have been explored for effective delivery [13]. Charge-based nonspecific electrostatic interactions are commonly used to load drugs in hydrogels, which are then released in the desired medium when the electrostatic interaction is screened by ions, or when the hydrogel is degraded [141]. Hydrophobic interactions can be used to load hydrophobic drugs in hydrogels, which are then released by the deterioration of strength and stability of hydrophilic hydrogels due to drug-polymer phase separation [142]. Cleavable to highly stable covalent linkages can be used to program the release of drugs in response to environmental cues. Cleavable covalent linkages range from small-molecule linkages (ester bonds, disulfide bonds, etc.) to macromolecular linkages (like peptide sequences cleavable by enzymes), which can be used to release drugs over a period. Conversely, stable covalent linkages include amide bonds (via carbodiimide chemistry), thiol-ene bonds, and metal-free click chemistry bonds that can be used for prolonged drug delivery by network degradation [143].

The diffusion and release of drugs from the hydrogels are controlled by the polymer network mesh size by steric interactions, which depends on crosslink density and external stimuli. The drug release process is dominated by diffusion when the mesh size is larger than the drug- where the larger the drug slower the diffusion [144]. The diffusivity (*D*) of the drug can be obtained from the viscosity (*η*) of the solution and the radius of the drug (*r*) using the following equation:

(1) $$\;D=\frac{RT}{6\pi \eta r}$$

where, *R* is the gas constant, and *T* is the absolute temperature. Moreover, the time required for drug diffusion depends on diffusion length, which can be estimated by *H*^2^/*D*, where *H* is the hydrogel thickness [145]. The effect of steric hindrance on drug diffusion becomes prominent when the mesh size is close to drug size, which leads to slower drug diffusion and extended release. When the mesh size is larger than the drug, the drug remains physically entrapped inside the hydrogel network, and to release the drug degradation of the network or increase the mesh size by swelling is necessary. The general release profiles of drugs from hydrogels can be described by the following equation:

(2) $$\frac{M_{t}}{M_{\infty }}=kt^{n}$$

where, *M_t_* is the mass of drug released at time *t*, *M_∞_* is the total mass of released drug, *k* is a kinetic constant, and *n* is the diffusional exponent [146]. When the drug release is dominated by Fickian diffusion, *n* = 0.5; whereas, when the drug release is dominated by surface erosion, *n* = 1.0. When more than one mechanism controls the release, the value of *n* is between 0.5 and 1.0 [147]. Therefore, a combination of the nature of interaction, hydrogel size, delivery routes, mesh size control drug release kinetics in a complex way. 

To endure the best performance in terms of drug release, the delivery system should be designed in accordance with the specific application and stimuli-release. In this context, the 4D printing allows for the fabrication of more sophisticated, stimuli-responsive and target specific drug delivery devices.

**Figure 10:**
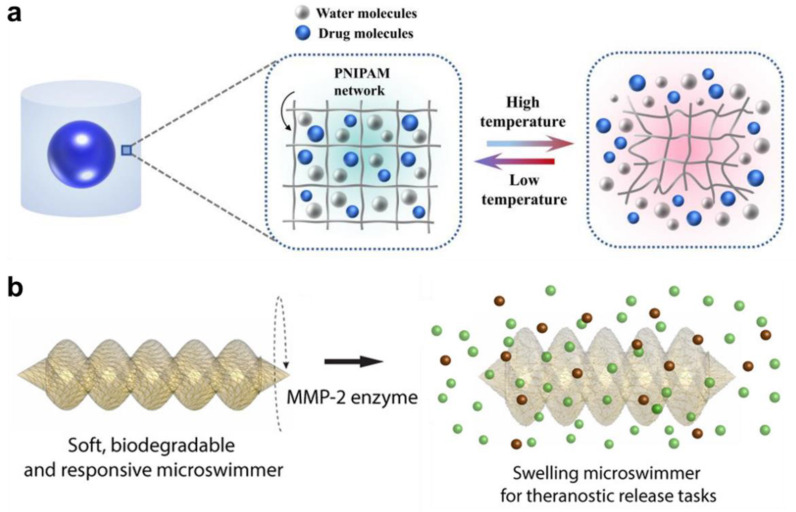
(**a**) Schematic of 4D printed core–shell (Brilliant blue—PNIPAM) capsule and their temperature responsive drug release. Reproduced with permission from [150]. Copyright © 2022 Elsevier. (**b**) Schematic of 4D printed Gelatin methacryloyl microbotic swimmers and their enzyme (Metalloproteinase 2) responsive drug (Fluorescein isothiocyanate–dextran) release. Reproduced with permission from [153]. Copyright © 2019. American Chemical Society.

Table 2 provides the summary of different 4D printed hydrogels demonstrated for stimuli-responsive drug delivery applications. These systems have been fabricated using different 3D printing methods including DIW, FDM, 2PP, SLA, and DLP. Wang et al. [148] fabricated methotrexate loaded pluronic diacrylate macromer and alginate-based hybrid hydrogel system (in the shape of square mesh) by DIW-4D printing using UV-curing. The hydrogels were demonstrated for aqueous calcium chloride (1% *w*/*v*) responsiveness (folding), which released around 80% of the drug over 12 h with a fast release profile for the first 6 h. On the other hand, Zhao et al. [149] fabricated heparin-loaded gelatine methacryloyl hydrogels (in the shape of strips on flat sheets) by DIW 4D printing and UV-curing, which folded into a tube-like structure when exposed to water and released around 70% of the drug over 30 h with a fast release profile for the first 8 h. Lately, Zu and co-workers [150] developed a core–shell capsule delivery system (Figure 10a) comprising a brilliant blue core and PNIPAM hydrogel shell by DIW 4D printing and UV-curing. These capsules are responsive to temperature and shrink to release almost 100% of the drug in 48 h with a fast release profile for the first 15 h. In a separate study, Melocchi et al. [151] fabricated caffeine-loaded I, U, and helix-shaped PVA hydrogel constructs by FDM 4D printing extruded at 180 °C. When exposed to water, the hydrogels showed swelling and shape transformation and released (fast release) almost 100% of the drug in the first 2 h.

Sitti and co-workers [152,153] have developed magnetically powered/controlled chitosan microswimmers by 2PP (Figure 10b), which can release embedded cargo and drug molecules (doxorubicin, and fluorescein isothiocyanate) by swelling and/or degradation in response to light or metalloproteinase enzyme. The system demonstrated a fast release profile (up to 1 min) under light followed by a steady release profile (up to 6 min) without light, whereas a fast release profile for the first 2 h in the presence of enzyme followed by a medium release up to 48 h. Such mobile microrobots have the potential for minimally invasive theranostic functions, which can be used to deliver drugs to hard-to-reach inner body sites.

**Figure 11:**
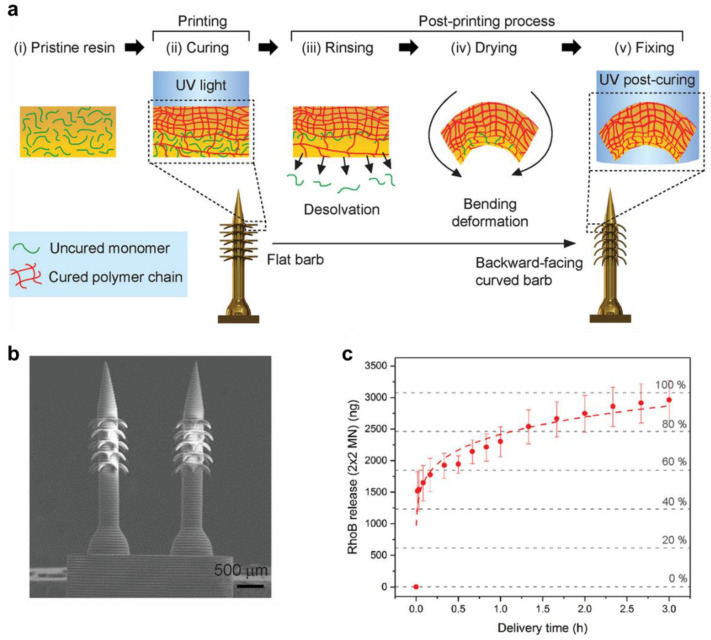
(**a**) Schematic of 4D printed PEGDA microneedle arrays with backward-Facing Barbs for enhanced tissue adhesion. (**b**) SEM images of 4D printed microneedle array (**c**) In vitro RhoB release kinetics of a barbed microneedle array. Reproduced with permission from [154]. Copyright © 2020 Wiley.

Conversely, Han et al. [154] fabricated poly(ethylene glycol) diacrylate microneedle array with the back facing curved barbs by 4D µSLA printing technique and post UV-curing, which released the drug rhodamine B when exposed to the stimuli phosphate-buffered saline (Figure 11). The arrays showed a fast release of the drug (50%) in just 1 min, followed by medium release for up to 2.5 h, and a slow release up to 3 h. The drug release from the array was also tested on a chicken breast skin-barrier model, which has potential for transdermal drug delivery applications. In a separate study, Fang et al. [155] developed stretchable 4-hydroxybutyl acrylate, and urethanepolyethylene glycol-polypropylene glycol nerve guide tubing by DLP 4D printing. These nerve guides were demonstrated for the controlled release of doxorubicin and ovalbumin protein under the external stimuli of a magnetic field. The doxorubicin showed fast release in the first 4 h, whereas ovalbumin showed fast release in the first 30 min, where both followed a medium release up to 50 h, and steady releases up to 75 h. The study demonstrated on-demand release of the drug by 4D constructs via magnetic field guided in vivo neuron regeneration. As a relatively new technology, four-dimensional (4D) printing is inspiring and promising to enable the creation and engineering of novel oral devices, the printing of medicines and on-demand fully customizable drug capsules with distinctive designs and drug release properties that have the advantage of autonomously controlling medication release in accordance with the actual physiological conditions.

## 5. Conclusions and Future Perspective

In conclusion, this review provides a broad overview of the ongoing research and current advances in formulations, mechanisms, and properties of stimuli-responsive smart hydrogels toward healthcare applications. Further, the basic concept, technical approaches, and challenges of the latest additive manufacturing technologies are also critically reviewed and discussed in correlation with the emerging 4D printed smart hydrogel carrier systems for the effective delivery of therapeutic drugs. Though there has been an explosion of articles on 3D printing of hydrogels, it appears that the trend for 4D printing of these smart hydrogels is still in its infancy. A broad set of versatile techniques and materials has been attempted and developed to integrate the two creative approaches of 3D printing and stimuli responsiveness at the micro-/nano scale for smart hydrogels. This comprehensive review demonstrates that there is huge room for 4D printing to be applied in many fields across the spectrum, and 4D printed smart hydrogels have the potential to bring outstanding breakthroughs for future healthcare.

Biomedical and healthcare applications, especially wound healing and drug delivery are areas where 4D printed smart hydrogels can have a great future impact. For instance, smart wound-healing bandages can be developed that can respond to the shape of the wound and healed area that may change with time. If the smart bandage can adjust the amount of drug released as well as shape shift to occupy the space of an unhealed wound through monitoring the cues from the skin structure, it can have a great effect on the healing process. The same healing mechanism can also occur for internal sutures in the body during major surgery. We foresee that in the next decades, 4D printed smart hydrogels will likely play a key role in various in vivo and in vitro tissue engineering and biomedical applications. Other potential applications for 4D printing in the medical field could be (i) design and fabrication of bio-mimetic functional structures such as microneedles with bioinspired backward-facing curved barbs for enhanced tissue adhesion (ii) printing consisting of self-folding protein, (iii) capsules with self-change drug release profiles in response to physiological cues as active control methods- a shift in the paradigm of smart controlled release of drugs and macromolecular active agents. The creation of 4D printed conformable tracheal airway splints, custom stents and small blood vessels (5–20 microns) will also be possible. These stents would be programmed to travel through the human body and open when they reached their destination, the airway splints could be expandable to allow the airways to grow with time. Additionally, the self-folding of 4D bio-printed vasculature can potentially result in the formation of blood vessels. The opportunity to develop personalized drugs, as well as disease and condition-dependent drug release for effective and safe therapies will be made possible through advancements in the design of multi-responsive bio-compatible and bio-responsive materials, innovation in additive manufacturing (AM) technology that allows multiple materials to be printed with precisely controlled heterogeneous microstructures, and the theoretical model to guide the selection of design parameters. 4D printing has created an amazing possibility to print products with full functionality built directly into the materials. However, the pathway to transform the novel ideas and possibilities to a functional product will be challenging and need to be explored further.

Beyond healthcare, there are new areas emerging for 4D printed smart hydrogels, which are the fashion industry, food industry and building/architecture. For instance, in the fashion industry, responsive cloths and accessories can be developed for healthcare settings such as aged care and disability sectors, where multi-stimuli responsive hydrogels can be embedded into clothes and supporting aids to not only monitor health conditions but also to respond in real time to the needs of the user. In the food industry, 4D printed foods are being developed to introduce innovation into food packaging, cooking, and consumption. For the building/architecture industry, 4D printed smart hydrogels can be embedded into buildings and infrastructures to keep the structures warm or cold depending on the external conditions, or to monitor bridges and roads for defects that can be corrected before fatal failures. 4D printed circuit boards are another interesting application that has a tremendous potential to pack more transistors in 3D with shape-shifting structures in response to real-time computing needs. These kinds of innovative applications would make the 4D printed materials transformed into 5D printed objects. As the active nature of 4D printed smart structures can be controlled by many external stimuli such as temperature, light, pH, humidity, magnetic and electrical, the combination of stimuli can be used to achieve multiple goals from the smart hydrogel, which is being dubbed ‘5D printing’.

However, there will be many challenges to overcome in the future to achieve the ideal level of control. These include materials challenges (responsiveness, cost, biocompatibility, sustainability), process challenges (processability, reliability, reproducibility, scalability), and challenges in achieving desirable properties for healthcare applications (in vivo and in vitro). Smart hydrogels are susceptible to interference from external stimuli found in the real world. Therefore, high-resolution and novel stimulation systems will need to be developed which can respond to complex multi-stimulation schemes in line with the many combinatorial and synergistic biological processes. When it comes to the future of 4D printed smart hydrogels, implementable, technical advancement is crucial for them to be practical in healthcare, particularly for drug delivery applications. A partial comprehension of a study field will not yield reliable procedures for fabricating 4D printed smart hydrogels. As a result, a deep and integrated grasp of material science, mechanical engineering, computational science and biomedical engineering is highly required for researchers developing a comprehensive strategy for producing and implementing 4D smart hydrogels for drug delivery. In the future, a new set of smart biomaterials with multi-stimuli responsiveness and bioinks for 4D printing originating from sustainable resources will need to be developed to fully expand its therapeutic opportunities.

## Figures and Tables

**Table 1:** pH in various tissues and cellular compartments [82].

Tissue/Cellular Compartment	pH
Stomach	1.0–3.0
Vagina	3.8–4.5
Late endosome	4.5–5.0
Upper small intestine	4.8–8.2
Inflamed tissue/wound	5.4–7.4
Early endosome	6.0–6.5
Tumor, extracellular	6.5–7.2
Colon	7.0–7.5
Blood	7.3–7.5

**Table 2:** Summary of emerging 4D printed hydrogels for drug delivery applications.

Materials	Printing Parameters	Crosslinking	Printed Shape Transformation	Drug & Loading Method	External Stimulus	Drug Release Profile	Ref.
Pluronic diacrylate macromer, and alginate	DIW: Nozzle diameter—400 µm; nozzle temperature—25 °C; print speed—15 mm/s; pressure—0.04 kPa; bed temperature—60 °C	UV curing	Square mesh to folded mesh	Methotrexate—Co-mixing	Ion (Calcium chloride)	Fast release up to 6 h followed by steady release up to 12 h	[148]
Gelatin methacryloyl	DIW: Nozzle diameter—210 µm; nozzle temperature—26 °C; print speed—20 mm/s	UV curing	Sheet to tubular	Heparin—Co-mixing	Solvent (Water)	Fast release up to 8 h followed by steady release up to 28 h	[149]
Poly(N-isopropylacrylamide)	DIW: Nozzle diameter—340 µm	UV curing	Expanding core–shell capsules	Brilliant blue and lemon yellow—Injection	Temperature (22 °C)	Fast release up to 15 h followed by medium release up to 48 h	[150]
Poly(vinyl alcohol)	FDM: Nozzle diameter—400 µm; nozzle temperature—180 °C; print speed—23 mm/s	-	I to U, U to I, and helix to extended conformation	Caffeine—Co-mixing	Solvent (Water)	Fast release up to 2 h followed by steady release up to 6 h	[151]
Methacrylamide Chitosan	2PP: Sub-micron resolution	-	Expandable microswimmers	Doxorubicin—Immersion	Light (UV 365 nm)	Fast release up to 1 min (light ON) followed by steady release up to 6 min (light OFF)	[152]
Gelatin methacryloyl	2PP: Sub-micron resolution	-	Expandable microswimmers	Fluorescein isothiocyanate—Immersion	Enzyme (Metallo-proteinase 2)	Fast release up to 2 h followed by medium release up to 48 h	[153]
Poly(ethylene glycol) diacrylate	µSLA: Layer thicknesss—50 µm	UV curing	Straight to backward-facing curved barbs in microneedle array	Rhodamine B—Immersion	Ion (Phosphate buffered saline)	Fast release in 1 min followed by medium release up to 2.5 h, and slow releases up to 3 h	[154]
4-hydroxybutyl acrylate, and urethanepolyethylene glycol-polypropylene glycol	DLP: Layer thicknesss—100 µm	UV curing	Stretchable nerve guide tubing	Doxorubicin, and ovalbumin—Immersion	Magnetic field (1 MHz)	Fast release in 4 h (doxorubicin) or 30 min (ovalbumin) followed by medium release up to 50 h, and steady releases up to 75 h	[155]

## Data Availability

Data sharing not applicable.
